# Breast lump as the initial presentation of metastatic uterine leiomyosarcoma: a case report and comprehensive literature review

**DOI:** 10.3325/cmj.2024.65.51

**Published:** 2024-02

**Authors:** Nermina Ibisevic, Kresimir Tomic, Alen Humackic, Zlatko Guzin, Blanka Lukic, Semir Vranic

**Affiliations:** 1 Department of Pathology, University of Sarajevo Clinical Center, Sarajevo, Bosnia and Herzegovina; 2 Department of Oncology, University Clinical Hospital Mostar, Mostar, Bosnia and Herzegovina; 3 Department of Radiology, Cantonal Hospital Dr. Safet Mujic, Mostar, Bosnia and Herzegovina; 4 Department of Surgery, Cantonal Hospital Dr. Safet Mujic, Mostar, Bosnia and Herzegovina; 5 College of Medicine, QU Health, Qatar University, Doha, Qatar; The first two authors contributed equally.

## Abstract

Uterine leiomyosarcoma (uLMS) is a rare but aggressive cancer with a high metastatic potential and an unfavorable prognosis. A 54-year-old woman with a history of uterine fibroids clinically presented with a painless, palpable left breast mass measuring 20 mm. A core biopsy of the breast mass demonstrated a cellular spindle cell neoplasm (a potentially malignant smooth muscle neoplasm; B4). A wide local breast-mass excision was performed, revealing grade-2 leiomyosarcoma. A re-review of the uterine fibroids revealed that the largest one (200 × 130 mm), initially diagnosed as symplastic leiomyoma, was morphologically identical to the breast lesion. Additional diagnostic work-up revealed multiple liver and pulmonary metastases with a suspected metastatic sclerotic lesion in the L3 projection. The patient was subsequently treated with chemotherapy protocol for metastatic uLMS. The latest follow-up in September 2023 confirmed stable disease. This case highlights the importance of considering unusual metastatic patterns when evaluating breast masses, particularly in patients with a history of non-specific uterine conditions. Comprehensive diagnostic work-up, including imaging and histopathologic examinations, is crucial for an accurate diagnosis of uLMS and appropriate treatment selection. Further studies are needed to better understand the underlying mechanisms and optimal management strategies for metastatic uLMS.

Uterine leiomyosarcoma (uLMS) is a cancer originating from mesenchymal tissue in the uterus. It is a relatively uncommon malignancy, accounting for only 2%-5% of all uterine cancers (1). The exact cause of uLMS remains unidentified, but genetic factors, hormone therapy, or radiation exposure may increase the likelihood of developing this type of cancer (1). The molecular mechanism underlying the development of uLMS remains unknown. However, the genomic and transcriptomic analysis showed substantial mutational heterogeneity, extensive DNA copy number alterations, and frequent inactivation of *TP53* and *RB1*, coupled with whole-genome duplication (2).

Patients may have non-specific symptoms or abnormal vaginal bleeding in the clinical presentation. The initial diagnostic workup involves a comprehensive evaluation by a gynecologist, including a transvaginal ultrasound and hysteroscopic endometrial biopsy, to assess suspicious changes. Imaging techniques such as magnetic resonance imaging (MRI) of the pelvis; computed tomography (CT) of the thorax, abdomen, and pelvis; or positron emission tomography (PET/CT) scan are used to assess the extent of the disease.

The histopathologic diagnosis of LMS relies on examining histologic features, including nuclear atypia, high mitotic index, and tumor necrosis (3). The most common histologic variant of uLMS is the spindle cell type, while epithelioid and myxoid leiomyosarcomas are rarer (4). Immunohistochemical analysis plays a pivotal role in refining the diagnosis of uLMS. All three variants exhibit varying degrees of immunohistochemical expression of smooth muscle markers such as desmin, h-caldesmon, smooth muscle actin (SMA), and histone deacetylase 8 (4).

The primary treatment modality for localized uLMS is hysterectomy with bilateral salpingo-oophorectomy (5). However, even in the early stages, >50% of patients experience relapses, which highlights the disease's aggressive nature (6). For advanced-stage disease, the recommended approach involves systemic therapy.

uLMS commonly metastasizes to the lungs, peritoneum, bone, and liver (7). Advanced-stage disease is treated with systemic therapy, including chemotherapy options such as doxorubicin, dacarbazine, trabectedin, and gemcitabine, either alone or in combination with docetaxel and pazopanib (8). Despite advancements in treatment, the five-year survival for uLMS ranges from 15% to 25% (7).

According to DeLair et al (9), the breast is a rare metastatic site, accounting for ~ 2% of all non-mammary malignancies. The same study found that the most common malignancies to metastasize to the breast, excluding hematologic malignancies, were gynecologic, gastrointestinal, and lung cancers (51%), melanoma (21%), and sarcoma (21%) (9). Among 14 metastatic gynecological cancers in the breast, the most common was ovarian serous carcinoma (10/14 cases) (9). Additionally, two cases of ovarian low-grade serous carcinoma and one of ovarian clear cell carcinoma were recorded. Regarding sarcomas that metastasized to breast tissue, uLMS were the most frequently observed (5/18 sarcomas) (9).

Breast metastases as an initial presentation of the metastatic uLMS are exceedingly rare. We present here such a case along with a comprehensive literature review.

## CASE REPORT

In May 2017, a 49-year-old patient presented with lower abdominal pain persisting for two weeks. The patient underwent conization of the cervix in 1997 due to cervical intraepithelial neoplasia III. Additionally, the patient’s aunt and daughter had been diagnosed with breast cancer. To investigate the cause of the abdominal pain, a multislice computed tomography (CT) of the abdomen and pelvis was performed. The CT revealed the presence of three distinct nodules within the uterus. The largest nodule measured 137 mm and had a lobulated appearance, while the other two measured 98 mm and 93 mm. Radiologic findings suggested the possibility of fibroids, characterized by multiple hypodense zones and suspicious cystic degeneration. However, alternative etiologies could not be ruled out. A lung x-ray was unremarkable. In July 2017, total abdominal hysterectomy and bilateral salpingo-oophorectomy were performed. The pathologic findings revealed the largest nodule to be an atypical (symplastic) leiomyoma, while the remaining two were classical leiomyomas. Based on these findings, routine gynecologic care was recommended.

**Figure 1:**
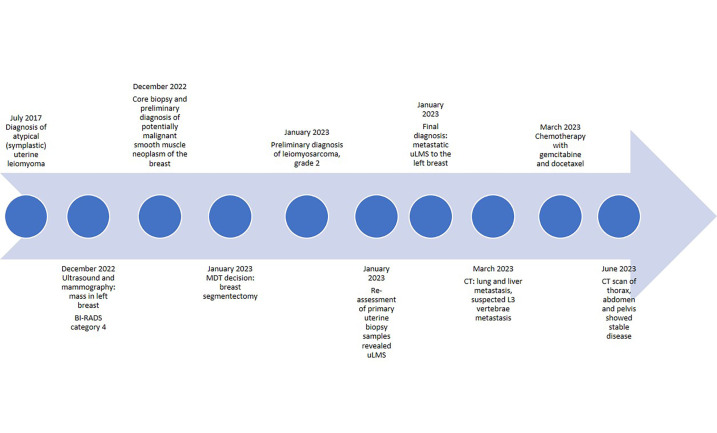
Mammography showed an oval lobulated well-circumscribed mass with a partly masked contour in the lower inner quadrant of the left breast. (**A**) Craniocaudal view; (**B**) mediolateral view.

**Figure 2:**
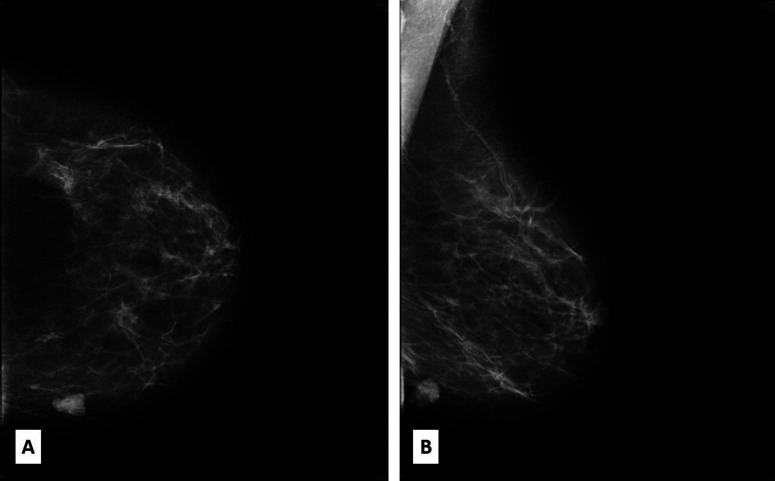
(**A**) Core biopsy revealing a spindle cell neoplasm in the breast (4 × ); (**B**) Surgical sample of metastatic leiomyosarcoma in the breast; note the presence of normal breast ducts adjacent to the well-circumscribed metastatic cancer (10 × ); (**C**) Primary uterine leiomyosarcoma with foci of necrosis (4 × ); (**D**) Spindle and pleomorphic cells with mitotic figures in primary uterine leiomyosarcoma (10 × ); All images represent hematoxylin and eosin stains.

In December 2022, during a routine ultrasound and mammography a suspicious finding was identified in the patient's left breast. The imaging revealed the presence of an oval-shaped mass with well-defined borders and macrolobulated features (Figure 1). The size of the mass was approximately 22 mm, and the mass was located in the lower medial quadrant of the left breast (Figure 1). These imaging findings were categorized as category 4 according to breast imaging-reporting and data system (BI-RADS). A subsequent core biopsy revealed a spindle cell neoplasm with moderate atypia and sporadic mitotic Figures (2/10 hpf) (Figure 2A). Based on the extended immunohistochemical analysis (cytokeratin pan, cytokeratin 5/6, S-100, leukocyte common antigen) and diffuse positivity for smooth muscle markers (SMA, smooth muscle myosin heavy chain, and desmin), a preliminary diagnosis of a potentially malignant smooth muscle neoplasm of the breast was made (B4 category). A multidisciplinary breast tumor board evaluated all the findings and recommended breast-conserving surgery with a segmentectomy, which were performed in January 2023.

Histopathologic examination of the resected specimen revealed similar findings observed in the core biopsy, but neoplastic cells exhibited marked atypia and pleomorphism with 6/20 hpf mitotic figures (Figure 2B). The proliferative index measured by Ki-67 was ~ 20%. Based on the morphology and immunohistochemical findings, the preliminary diagnosis was grade-2 leiomyosarcoma. Given the patient's previous pathologic findings from 2017 and the exceptional rarity of malignant smooth muscle neoplasms in the breast, the possibility of a uterine origin of the tumor was re-considered. To further investigate the nature of the tumor, the previously operated symplastic leiomyoma of the uterus was compared with the newly removed breast tumor. A re-assessment of the primary uterine biopsy samples from 2017 revealed that the largest nodule (200 × 130 mm) exhibited all the morphologic features (severe pleomorphism/atypia, mitotic Figures [12/10 hpf mitoses], and the presence of tumor cell necrosis) of uLMS (Figure 2C-D). Consequently, the final diagnosis was metastatic uLMS to the breast.

**Figure 3:**
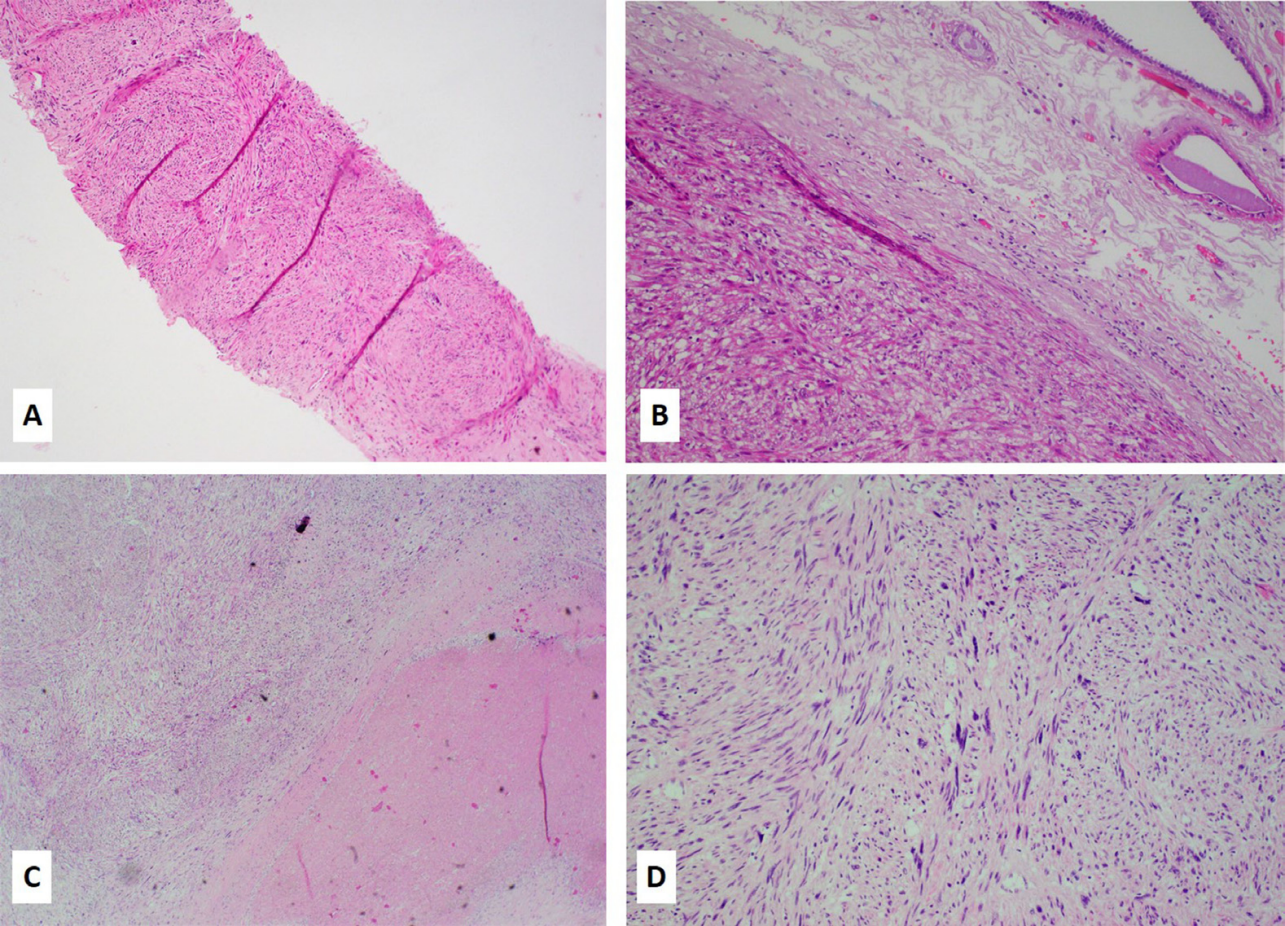
Bilateral metastasis in the pulmonary parenchyma of primary uterine leiomyosarcoma.

**Figure 4:**
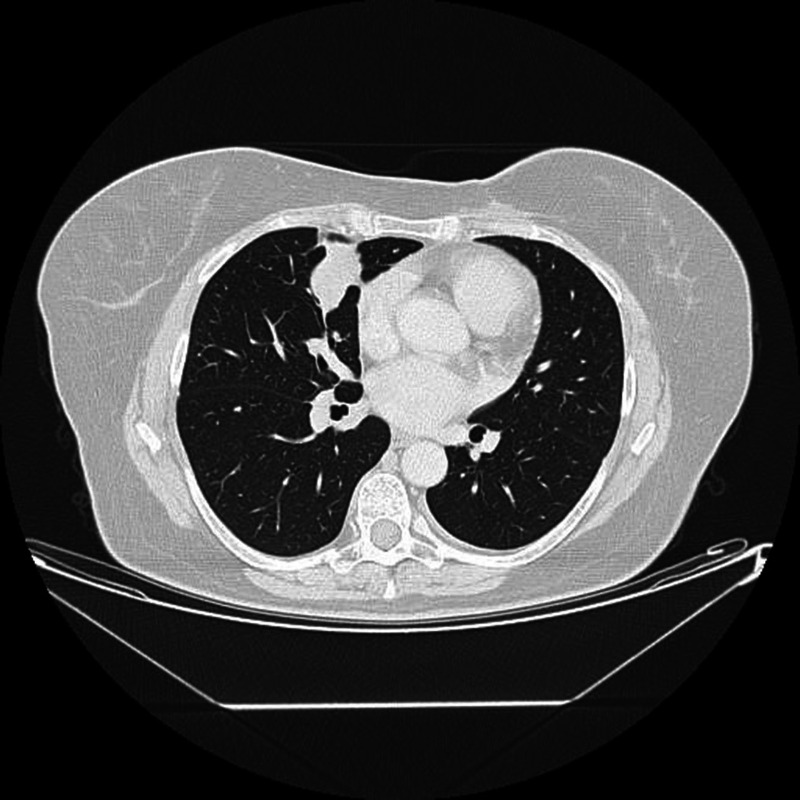
The timeline of the diagnostic tests and treatments. BI-RADS – breast imaging-reporting and data system; MDT – multidisciplinary team; uLMS – uterine leiomyosarcoma; CT – computed tomography.

In March 2023, thoracic multi-slice computer tomography (MSCT) revealed bilateral metastases in the pulmonary parenchyma and subpleural regions. The largest metastatic lesion, measuring 40 mm, was in the right middle lobe (Figure 3). Furthermore, abdominal and pelvic MSCT showed multiple liver metastases, with the largest measuring 72 mm in the left lobe (not shown). Additionally, MSCT revealed an oval sclerotic lesion measuring 11.5 mm in the L3 vertebra of the spinal column, with suspicion of metastasis from uLMS (not shown). To further evaluate bone metastases, bone scintigraphy was recommended. In March 2023, the patient initiated chemotherapy with a combination of gemcitabine and docetaxel. Since then, the patient completed nine cycles of chemotherapy with preventive granulocyte colony-stimulating factor filgrastim. She tolerated the chemotherapy well, with hair loss being the only notable side effect. A follow-up CT scan of the thorax, abdomen, and pelvis made in June 2023 showed stable disease. A control CT scan performed in September 2023 confirmed stable disease. The timeline of diagnostic tests and treatments is shown in Figure 4.

## DISCUSSION

We present a rare case of a patient with metastatic uLMS to the breast, initially misdiagnosed as atypical leiomyoma. Primary LMS of the breast is an extremely rare disease, with only 54 cases described in the literature (10). Clinically, it presents as a large, painless mass in the breast. In our patient, the breast mass was painless and small. The pathologic findings after the segmentectomy of the left breast showed metastases originating from the uLMS previously misdiagnosed as atypical leiomyoma. Compared with leiomyoma, uLMS is a rare, aggressive tumor with a high preponderance of distant metastases to the lungs, liver, and bone. Distinguishing between uLMS and leiomyoma can be challenging due to clinically similar symptoms, such as increased uterine size, abdominal pain, and vaginal bleeding (11). This case emphasizes the significance of interdisciplinary collaboration between gynecologists, oncologists, pathologists, and radiologists to accurately diagnose and manage uLMS. Despite clear diagnostic criteria, the distinction between uLMS and symplastic leiomyoma can be challenging in some cases (12). However, the microscopic tumor cell necrosis and ≥ ten mitoses/hpf indicate uLMS, not symplastic (atypical) leiomyoma (13).

To our knowledge, only eleven cases of uLMS with breast metastases have been documented in the medical literature, along with their clinical presentations, imaging findings, immunohistochemical profile, treatment modalities, time from uLMS diagnosis to breast metastasis, and overall survival. The details of these cases are summarized in Table 1. One such case involved a patient diagnosed with metastatic uLMS in the left breast three years after hysterectomy due to a fibroid uterus (14). A retrospective pathology review revealed that the sarcoma originated from a previously benign fibroid (14). Interestingly, similar to our patient, the diagnosis changed from atypical (symplastic) leiomyoma to uLMS.

**Table 1:** Summary of cases with metastatic uterine leiomyosarcoma in the breast

Case	Authors	Age at diagnosis	Clinical presentation	Imaging findings	Positive immunohistochemistry results	Treatment	Time from uLMS diagnosis to breast metastasis (months)	Overall survival (months)
1	Lin et al (15)	44	Bilateral breast lumps	Lung Pleural surface Liver Intrabdominal	Desmin Vimentin NSE (weakly positive)	Hysterectomy Right salpingo-oophorectomy	132	Alive at publication
2	Madigan et al (21)	60	Right breast mass	Subcutaneous metastasis of the neck and axilla Lung	Muscle-specific actin	TAH Chemotherapy with doxorubicin and ifosfamide	120	
3	Sibartie et al (14)	56	Left breast mass	Lung Liver Bone	Not reported	Hysterectomy	36	40
4	Hsiao et al (22)	62	Left breast mass	Lung Liver Bone	Desmin SMA	TAH and BSO Chemotherapy with ifosfamide, etoposide and carboplatin	72	17
5	Yasuhara et al (23)	48	Left breast mass	No evidence of metastasis	SMA Vimentin	TAH and BSO	Simultaneously detected uLMS	36
6	Pappa et al (24)	58	Left breast mass	Lung Liver Kidneys Adrenal glands	SMA Desmin Vimentin HHF35 (diffuse moderate positivity)	TAH and BSO Chemotherapy Radiotherapy	18	Not reported
7	Noronha et al (25)	74	Left breast mass	Extensive pelvic disease Lung	Desmin SMA Vimentin p63 (focally positive)	Not reported	Not reported	Not reported
8	Vasan et al (26)	46	Painless bilateral breast masses	Lung Liver Bone	SMA Desmin HHF35	Active surveillance according to the patient's wishes	Simultaneously detected uLMS	Not reported
9	Tirumani et al (7)	Not reported	Not reported	Not reported	Not reported	Not reported	Not reported	Not reported
10	Karaman et al (27)	56	Breast mass	Lung	Not reported	TAH and BSO Chemotherapy with ifosfamide, mesna and adriamycin	24	Alive at publication
11	Colón et al (28)	51	Right breast mass	Not reported	SMA Desmin Vimentin Caldesmon	Hysterectomy Salpingo-oophorectomy	120	Not reported

*Abbreviations: uLMS – uterine leiomyosarcoma; TAH – total hysterectomy; BSO – bilateral salpingo-oophorectomy; SMA – smooth muscle actin; NSE – neuron-specific enolase.

In our patient, five years passed from the initial surgery of the uterus to the appearance of distant metastases. The literature describes cases of uLMS that metastasized to the breast twelve years after the initial diagnosis (15) (Table 1). Distant metastases were verified in 81% of uLMS patients after seven months of clinical follow-up (7). Such a rapid relapse of the disease in many patients best illustrates how aggressive the course of uLMS is. Clinical prognostic factors for the development of metastases are age over 50 years, disease stage, and the local recurrence of disease (7). Our patient was 49 years old at the time of surgery, FIGO stage IB, without local recurrence. We believe all three favorable clinical factors are the reason for her long five-year period without metastasis. Among the histopathologic parameters, negative prognostic factors for the development of metastases are serosal involvement and necrosis (7). In our patient, the serous infiltration status was unknown, and the sample had necrosis in the primary uLMS.

The uncommon presence of biomarkers such as microsatellite instability, high tumor mutational burden, and the lack of targetable genomic alterations restrict a personalized approach and treatment of patients with uLMS (16). Patients with cancers that have metastasized to the breast may benefit from personalized immunotherapy and targeted therapies due to considerable heterogeneity in biomarkers (17). Considering the positive family history of breast cancer, conducting next-generation sequencing testing would be advisable to enable a potential personalized treatment approach for our patient. Specifically, 5% of uLMS patients exhibit a *BRCA2* mutation, and therapy with PARP inhibitors has shown sustained partial responses in these cases (18).

The standard approach for treating localized uLMS involves surgical intervention as the primary treatment modality (5). After surgery, adjuvant chemotherapy or pelvic radiation are sometimes considered; the decision depends on the risk factors for disease relapse. In addition to the these therapeutic options, the preferred chemotherapy regimen in metastatic settings includes doxorubicin as a monotherapy or in combination with ifosfamide or dacarbazine (5). Additionally, the combination of gemcitabine and docetaxel can also be employed (5). For metastatic disease, two phase-II clinical studies have shown the benefit of chemotherapy with gemcitabine and docetaxel in first- and second-line chemotherapy for uLMS (19,20). A phase-II study evaluating first-line chemotherapy with gemcitabine plus docetaxel for uLMS confirmed a high response rate of 37% (20). The major toxicity was myelosuppression, with 17% of patients experiencing grade-3 and grade-4 neutropenia and 14% of patients experiencing grade-3 and grade-4 thrombocytopenia. After completing four cycles of chemotherapy with gemcitabine plus docetaxel, our patient's radiologic evaluation revealed stable disease, and no hematological toxicity was observed up to the moment of writing this report.

This case highlights the importance of considering unusual metastatic patterns when evaluating breast masses, particularly in patients with a history of non-specific uterine conditions. Comprehensive diagnostic work-up, including imaging and histopathologic examinations, is crucial for accurate diagnosis and appropriate treatment selection. Further studies are needed to better understand the underlying mechanisms and optimal management strategies for metastatic uLMS.

## References

[1] Byar KL Fredericks T Uterine leiomyosarcoma. J Adv Pract Oncol 2022 13 70 6 10.6004/jadpro.2022.13.1.6 35173990 PMC8805803

[2] Chudasama P Mughal SS Sanders MA Hubschmann D Chung I Deeg KI Integrative genomic and transcriptomic analysis of leiomyosarcoma. Nat Commun 2018 9 144 10.1038/s41467-017-02602-0 29321523 PMC5762758

[3] Gao T Finkelman BS Ban Y Li Y Yin P Bulun SE Integrated histologic and molecular analysis of uterine leiomyosarcoma and 2 benign variants with nuclear atypia. Cancer Sci 2021 112 2046 59 10.1111/cas.14775 33338329 PMC8088951

[4] Prat J Mbatani. Uterine sarcomas. Int J Gynaecol Obstet 2015 131 Suppl 2 S105 10 10.1016/j.ijgo.2015.06.006 26433666

[5] NCCN Clinical Practice Guidelines in Oncology, Uterine Neoplasms Version 2.2023 — April 28, 2023.

[6] Major FJ Blessing JA Silverberg SG Morrow CP Creasman WT Currie JL Prognostic factors in early-stage uterine sarcoma. A Gynecologic Oncology Group study. Cancer 1993 71 4 Suppl 1702 9 10.1002/cncr.2820710440 8381710

[7] Tirumani SH Deaver P Shinagare AB Tirumani H Hornick JL George S Metastatic pattern of uterine leiomyosarcoma: retrospective analysis of the predictors and outcome in 113 patients. J Gynecol Oncol 2014 25 306 12 10.3802/jgo.2014.25.4.306 25142630 PMC4195301

[8] Gronchi A Miah AB Dei Tos AP Abecassis N Bajpai J Bauer S Soft tissue and visceral sarcomas: ESMO-EURACAN-GENTURIS Clinical Practice Guidelines for diagnosis, treatment and follow-up Ann Oncol 2021 32 1348 65 10.1016/j.annonc.2021.07.006 34303806

[9] DeLair DF Corben AD Catalano JP Vallejo CE Brogi E Tan LK Non-mammary metastases to the breast and axilla: a study of 85 cases. Mod Pathol 2013 26 343 9 10.1038/modpathol.2012.191 23174933

[10] Ilyas MIMNS Xiao PQ Breast leiomyosarcoma: a systematic review and recommendations for management. Int Surg 2019 104 196 202 10.9738/INTSURG-D-15-00183.1

[11] Smith J Zawaideh JP Sahin H Freeman S Bolton H Addley HC Differentiating uterine sarcoma from leiomyoma: BET(1)T(2)ER Check! Br J Radiol 2021 94 20201332 10.1259/bjr.20201332 33684303 PMC9327746

[12] Pushpalatha K Kumar S Dinda AK Sharma JB Symplastic leiomyoma of uterus–a clinico-pathological dilemma BMJ Case Rep 20112011 Nov 15:2011:bcr0920114835 10.1136/bcr.09.2011.4835 22674600 PMC3229402

[13] Leiomyoma-general 2023. Available from: https://www.pathologyoutlines.com/topic/uterusleiomyoma.html. Accessed: February 2, 2024.

[14] Sibartie S Larkin JO Lee G Fitzgibbon J O’Reilly S Richardson D Metastatic uterine leiomyosarcoma presenting as a breast lump. Ir J Med Sci 2011 180 889 91 10.1007/s11845-009-0286-8 19184604

[15] Lin CH Yeh CN Chen MF Breast metastasis from uterine leiomyosarcoma: a case report. Arch Gynecol Obstet 2003 267 233 5 10.1007/s00404-002-0309-4 12592426

[16] Treatment and prognosis of uterine leiomyosarcom. UpToDate. 2021. Available from: https://www.uptodate.com/contents/treatment-and-prognosis-of-uterine-leiomyosarcoma/print. Accessed: February 2, 2024.

[17] Vranic S Senarathne W Stafford P Poorman K Pockaj BA Gatalica Z Biomarkers of targeted therapy and immuno-oncology in cancers metastatic to the breast. Appl Immunohistochem Mol Morphol 2020 28 661 8 10.1097/PAI.0000000000000808 31517642 PMC7664953

[18] Hensley ML Chavan SS Solit DB Murali R Soslow R Chiang S Genomic landscape of uterine sarcomas defined through prospective clinical sequencing. Clin Cancer Res 2020 26 3881 8 10.1158/1078-0432.CCR-19-3959 32299819 PMC7367750

[19] Hensley ML Blessing JA Degeest K Abulafia O Rose PG Homesley HD Fixed-dose rate gemcitabine plus docetaxel as second-line therapy for metastatic uterine leiomyosarcoma: a Gynecologic Oncology Group phase II study. Gynecol Oncol 2008 109 323 8 10.1016/j.ygyno.2008.02.024 18394689 PMC2692926

[20] Hensley ML Blessing JA Mannel R Rose PG Fixed-dose rate gemcitabine plus docetaxel as first-line therapy for metastatic uterine leiomyosarcoma: a Gynecologic Oncology Group phase II trial. Gynecol Oncol 2008 109 329 34 10.1016/j.ygyno.2008.03.010 18534250 PMC2504727

[21] Madigan MN Dempsey PJ Krishnamurthy S Ultrasound-guided fine needle aspiration cytodiagnosis of leiomyosarcoma metastatic to the breast. A case report. Acta Cytol 2003 47 783 6 10.1159/000326606 14526679

[22] Hsiao HH Liu YC Hou MF Lin SF Uterine leiomyosarcoma metastasis to the breast. Eur J Gynaecol Oncol 2008 29 191 2 18459564

[23] Yasuhara Y Mikami Y Ishiguro S Metastatic breast carcinoma identified in a uterine leiomyosarcoma. Pathol Int 2008 58 317 21 10.1111/j.1440-1827.2008.02230.x 18429832

[24] Pappa L Zagorianakou N Kitsiou E Sintou-Mantela E Bafa M Malamnou-Mitsi V Breast metastasis from uterine leiomyosarcoma diagnosed by fine needle aspiration: a case report. Acta Cytol 2008 52 485 9 10.1159/000325559 18702371

[25] Noronha YS Apple SK Uterine leiomyosarcoma presenting as breast metastasis. Breast J 2013 19 107 9 10.1111/tbj.12054 23167950

[26] Vasan N Saglam O Killelea BK Metastatic leiomyosarcoma presenting as bilateral, multifocal breast masses BMJ Case Rep 20122012: bcr2012007188 10.1136/bcr-2012-007188 23220834 PMC4544926

[27] Karaman SHH Uterine leiomyosarcoma with breast metastasis: a case report. Journal of Cancer Science and Clinical Therapeutics. 2018 2 100 1 10.26502/jcsct.5079014

[28] Colon E Metastasis of uterine leiomyosarcoma to the breast: medical and histopathological criteria. Case Rep Pathol 2020 2020 8037646 10.1155/2020/8037646 33381340 PMC7762671

